# Systematic literature review and trial-level meta-analysis of aromatase inhibitors vs tamoxifen in patients with HR+/HER2− early breast cancer

**DOI:** 10.1016/j.breast.2025.104429

**Published:** 2025-03-05

**Authors:** Wolfgang Janni, Michael Untch, Nadia Harbeck, Joseph Gligorov, William Jacot, Stephen Chia, Jean-François Boileau, Subhajit Gupta, Namita Mishra, Murat Akdere, Andriy Danyliv, Giuseppe Curigliano

**Affiliations:** a Department of Gynecology and Obstetrics, Ulm University, Ulm, Germany; b Interdisciplinary Breast Cancer Center, Helios Klinikum Berlin-Buch, Berlin, Germany; c Breast Center, Department of Obstetrics and Gynecology, University Hospital of Munich Ludwig Maximilian, Munich, Germany; d Institut Universitaire de Cancérologie, AP-HP Sorbonne Université, Paris, France; e Institut du Cancer de Montpellier Val d'Aurelle, Montpellier University, INSERM, Montpellier, U1194, France; f BC Cancer Research Centre, Vancouver, BC, Canada; g Montreal Jewish General Hospital, Segal Cancer Centre, McGill University, Montreal, QC, Canada; h Novartis Healthcare Private Limited, Hyderabad, Telangana, India; i Novartis Pharma AG, Basel, Switzerland; j European Institute of Oncology IRCCS, Milano, Italy; k Department of Oncology and Hemato-Oncology, University of Milano, Milano, Italy

**Keywords:** Meta-analysis, HR+/HER2− early breast cancer, Adjuvant endocrine therapy, Tamoxifen, Aromatase inhibitor

## Abstract

**Background:**

Current standard of care for patients with HR+/HER2− early breast cancer (EBC) includes adjuvant endocrine therapy with an aromatase inhibitor (AI) or tamoxifen (TAM). We present a trial-level meta-analysis on efficacy of AI vs TAM in patients with HR+/HER2− EBC.

**Methods:**

A systematic literature review was conducted using key medical literature databases (eg, PubMed; inception to October 2023) and data from conferences (to December 2023). Phase 3 randomized controlled trials (RCTs) that had ≥80 % of patients with HR+/HER2− EBC (or available subgroup data) and reported a disease-free survival (DFS) hazard ratio for AI vs TAM were included in the meta-analysis, regardless of menopausal status and ovarian function suppression (OFS) use. The generic invariance method was used to calculate a pooled effect estimate of DFS hazard ratios and 95 % CIs. A base-case analysis (all RCTs) and scenario analyses for NSAI-only, premenopausal, and postmenopausal RCTs were conducted.

**Results:**

Five RCTs were identified for inclusion in the meta-analysis. In the base-case analysis, DFS significantly favored AI ± OFS vs TAM ± OFS (pooled hazard ratio, 0.68; 95 % CI, 0.61–0.76; *P* < .0001). Results from scenario analyses were consistent with the base case; NSAI-only (pooled hazard ratio, 0.68; 95 % CI, 0.59–0.78; *P* < .0001), premenopausal (pooled hazard ratio, 0.65; 95 % CI, 0.56–0.76; *P* < .0001), and postmenopausal (pooled hazard ratio, 0.72; 95 % CI, 0.61–0.86; *P* = .001) RCTs favored AI ± OFS over TAM ± OFS.

**Conclusions:**

This trial-level meta-analysis demonstrated a significant DFS benefit with AI vs TAM for patients with HR+/HER2− EBC, which was more pronounced in premenopausal women.

## Highlights

• Standard of care for HR+/HER2− early breast cancer includes adjuvant tamoxifen (TAM) or aromatase inhibitor (AI).
• A meta-analysis of randomized controlled trials (RCTs) was conducted to compare disease-free survival with adjuvant TAM and AI.
• A total of 5 RCTs (constituting the base case) were identified and included in the meta-analysis.
• In addition to a base case analysis, scenario analysis included nonsteroidal AI, premenopausal, and postmenopausal RCTs.
• The results showed a significant benefit with AI over TAM in the base case and all scenarios analyzed.

## 1 Introduction

Hormone receptor–positive, human epidermal growth factor receptor 2–negative (HR+/HER2−) breast cancer is the most common subtype of early breast cancer (EBC) [1]. Standard of care for patients with HR+/HER2− EBC includes at least 5 years of adjuvant endocrine therapy (ET) [1,2]. Historically, tamoxifen (TAM) was the first ET widely used for adjuvant therapy; subsequent introduction and approval of aromatase inhibitors (AIs) such as anastrozole, letrozole, and exemestane eventually led to treatment guidelines recommending an AI or TAM for postmenopausal patients and an AI + ovarian function suppression (OFS) or TAM ± OFS for premenopausal patients with HR+/HER2− EBC.

The choice of an AI or TAM for adjuvant ET depends on factors such as risk of recurrence, disease features, menopausal status, and patient preference. Real-world studies have shown varying treatment patterns between the two classes depending on region, with AI generally constituting the majority of ET initiations [[3], [4], [5], [6]]. There are also variations in treatment patterns of AIs and TAM depending on menopausal status, with a higher proportion of premenopausal patients being treated with TAM. However, there is a trend of increasing AI use in premenopausal patients over time, as well as an increased use of OFS, particularly among patients with higher risk of recurrence [7].

The choice of AI vs TAM has been informed by clinical trials comparing the efficacy of the two classes in patients with HR + EBC. In postmenopausal patients with HR+ disease, an AI was associated with lower rates of recurrence compared with TAM [8]. Among premenopausal patients, clinical trials have suggested that an AI + OFS was more beneficial than TAM + OFS or TAM alone [9,10]. Additionally, meta-analyses using patient-level data across randomized clinical trials (RCTs) in patients with HR+ EBC have supported AIs as more effective than TAM for reducing recurrences in postmenopausal patients [11]. Among premenopausal patients, a similar meta-analysis demonstrated that an AI + OFS was more effective than TAM + OFS in reducing the risk of recurrence [12]. These meta-analyses included all patients with HR+ EBC regardless of HER2 status and therefore included those with HER2+ and HER2− disease [11,12]. Here, we present a trial-level meta-analysis of AI ± OFS vs TAM ± OFS focusing specifically on patients with HR+/HER2− EBC.

## 2 Materials and methods

### 2.1 Systematic literature review

Trials for the meta-analysis were identified through a systematic literature review that was consistent with PRISMA criteria (**Supplementary Methods** and Supplementary Fig. 1). Key medical literature databases (eg, Embase, Medline [via PubMed], Cochrane) were searched from their inception to October 2023, and data from key conferences were searched to December 2023. Key inclusion criteria were randomized clinical or noninterventional trials studying HR+/HER2− EBC in patients ≥18 years of age and published in English. Studies were required to have ≥80 % of patients with HR+/HER2− EBC. If the trials had <80 % of patients with HR+/HER2− EBC, separate data for this subgroup was required. Studies were included regardless of patients’ menopausal status or OFS use. Titles and abstracts of studies from the search were reviewed, followed by full text screening for inclusion in the analysis. Studies were screened by two independent reviewers, and any discrepancy between reviewers was resolved by a third independent reviewer.

### 2.2 Meta-analyses

For the meta-analysis, only phase 3 RCTs identified through the systematic literature review that compared an AI vs TAM and reported a disease-free survival (DFS) hazard ratio were included. RCTs were analyzed, and data categories were extracted, including DFS hazard ratio, treatment arms, menopausal status, median follow-up, and length of treatment. The generic invariance method was used to obtain a pooled effect-estimate hazard ratio and its 95 % CI for DFS, which was calculated as a weighted average of the intervention effects estimated in each individual trial. A fixed effect model (FEM) and randomized effect model (REM) were used to estimate the effect size. *I*^2^ was used to assess heterogeneity in the trials, and a funnel plot was used to assess publication bias in the effect size.

A base case analysis was performed that included all studies meeting inclusion criteria for the meta-analysis. Additional scenario analyses included RCTs that investigated premenopausal women only, postmenopausal women only, and nonsteroidal aromatase inhibitors (NSAIs) only in order to assess potential differences due to class of AI. An additional sensitivity analysis was performed to test the consistency of outcomes when data from an individual RCT of interest (pooled SOFT and TEXT trial data, since individual TEXT data were not available) were added to all existing studies in the base case and to those that investigated only premenopausal women. This sensitivity analysis was carried out to assess whether the results were consistent with those in which data from TEXT were excluded (and SOFT individual data were included).

## 3 Results

### 3.1 Systematic literature review

A total of 136 reports were returned by the initial systematic literature search, from which five RCTs (SOFT, HOBOE, BIG 1–98, N-SAS BC03, and NCT01352091) were identified for inclusion in the meta-analysis (Table 1). The HOBOE trial included two arms of AI (letrozole + OFS; or zoledronic acid + letrozole + OFS) vs TAM + OFS, and each arm of AI was compared individually. In the NCT01352091 trial, patients started on 2–3 years of TAM before being randomized to anastrozole + goserelin or TAM (for a total treatment duration of 5 years). Of note, the TEXT trial was not included in the meta-analysis because available published data from TEXT were pooled with data from SOFT; thus, TEXT did not qualify for inclusion in the systematic literature review. A sensitivity analysis that included the pooled TEXT and SOFT data was performed to analyze any impact. A funnel plot of the five RCTs included in the meta-analysis did not show any publication bias for effect size (Supplemental Fig. 2).

**Table 1:** Trials identified by systematic literature search for inclusion in meta-analysis.

Trial	Study	Intervention	Comparator	Time of assessment, years^a^	Median follow-up, years	Menopausal status
SOFT	Francis 2023	Exemestane + OFS (n = 1014)	TAM (n = 1018)	12	12	Premenopausal
HOBOE	Gravina 2022	Letrozole + OFS (n = 356)	TAM + OFS (n = 354)	8	8.6	Premenopausal
HOBOE	Gravina 2022	Zoledronic acid + letrozole + OFS (n = 355)	TAM + OFS (n = 354)	8	8.6	Premenopausal
BIG 1-98	Filho 2015	Letrozole (n = 1782)	TAM (n = 1751)	4	8.1^b^	Postmenopausal
N-SAS BC03	Aihara 2010	Anastrozole (n = 165)	TAM (n = 164)	3	3.5 (42 months)	Postmenopausal
NCT01352091	Li 2019	TAM → goserelin + anastrozole (n = 33)^c^	TAM (n = 29)	5	2.8 (34 months)	Premenopausal

OFS, ovarian function suppression; TAM, tamoxifen.

a After start of adjuvant ET.

b For the time point selected, median follow-up was 51 months, while 8.1 years is the overall follow-up of the study.

c The symbol → denotes treatment switching.

Among the five RCTs, three focused on patients with premenopausal disease, while two studied those with postmenopausal disease (Table 1). NSAI (in four RCTs) was the most common AI comparator to TAM. The five trials included data from a total of 7138 patients. Median follow-up time for all RCTs ranged between 2.8 and 12 years: SOFT (12 years), HOBOE (8.6 years for both AI comparisons), BIG 1–98 (8.1 years), N-SAS BC03 (3.5 years), and NCT01352091 (2.8 years). Additional study details and risk of bias assessment are presented in Supplemental Table 1 and Supplemental Table 2.

### 3.2 Meta-analyses: base case and scenario analyses

**Fig. 1:**
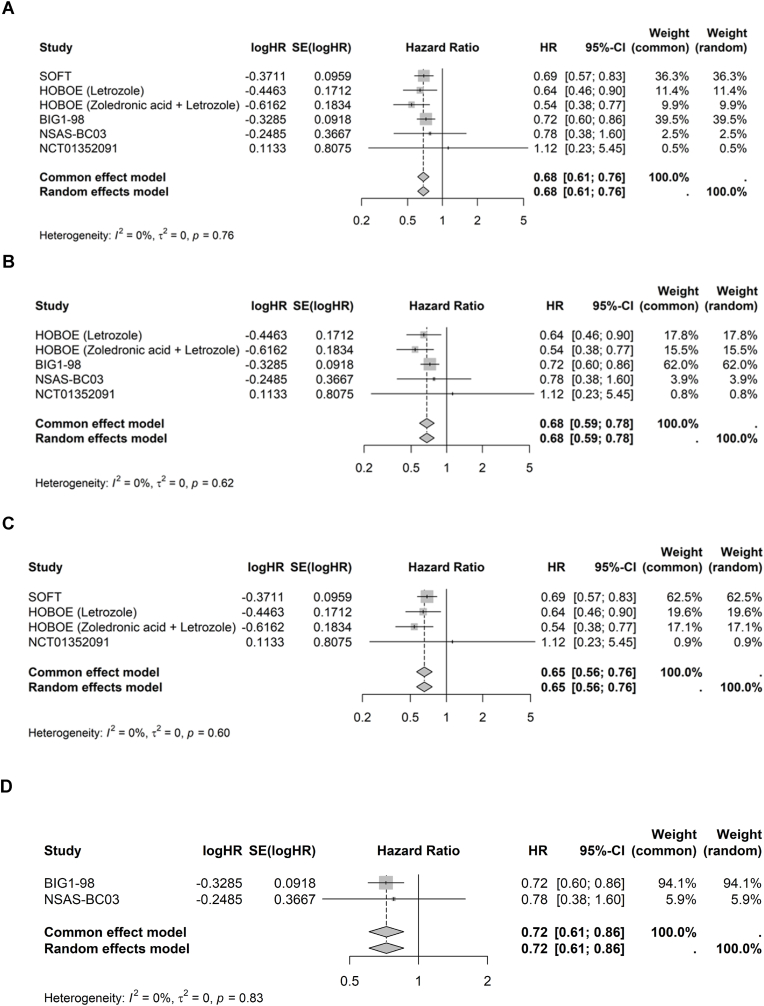
Forest plot of all studies (A), NSAI studies (B), premenopausal patients (C), postmenopausal patients (D).

In the base case and scenario analyses, *I*^2^ = 0 indicated no heterogeneity. Therefore, the outcomes of FEM and REM analyses were identical. Analysis of the base case (all five RCTs) demonstrated that DFS significantly favored AI ± OFS compared with TAM ± OFS, with a pooled hazard ratio of 0.68; 95 % CI, 0.61–0.76; *P* < .0001 (Fig. 1A). This result corresponds to a 32 % reduction in risk of recurrence or death. Scenario analysis that included only the studies that specifically evaluated NSAI (HOBOE, BIG 1–98, N-SAS BC03, and NCT01352091) was consistent with the base case, with DFS significantly favoring NSAI ± OFS over TAM ± OFS (pooled hazard ratio, 0.68; 95 % CI, 0.59–0.78; *P* < .0001) (Fig. 1B).

Among the studies that included only premenopausal women (SOFT, HOBOE, and NCT01352091), DFS significantly favored AI + OFS vs TAM ± OFS (pooled hazard ratio, 0.65; 95 % CI, 0.56–0.76; *P* < .0001) (Fig. 1C). A DFS benefit was also observed with AI vs TAM among studies that included only postmenopausal patients (BIG 1–98 and N-SAS BC03), with a pooled hazard ratio of 0.72; 95 % CI, 0.61–0.86; *P* = .001 (Fig. 1D).

### 3.3 Sensitivity analyses

When data from the TEXT trial were included (pooled with data from the SOFT trial) with all base case studies, the results remained consistent, favoring AI ± OFS. Similar results were observed for both fixed effects (pooled hazard ratio, 0.74; 95 % CI, 0.67–0.81) and random effects models (pooled hazard ratio, 0.72; 95 % CI, 0.64–0.82), with a heterogeneity of *I*^2^ = 2 % (Table 2). This was also observed when TEXT was included in the scenario analysis of studies that included only premenopausal patients (for both fixed and random effects models: pooled hazard ratio, 0.77; 95 % CI, 0.69–0.85); heterogeneity *I*^2^ = 0.

**Table 2:** Sensitivity analysis: Pooled SOFT and TEXT trial data.

Scenarios	Heterogeneity, %	Pooled hazard ratio	95 % CI
Fixed effects: All studies	2	0.74	0.67–0.81
Random effects: All studies		0.72	0.64–0.82
Fixed effects: Premenopausal studies	0	0.77	0.69–0.85
Random effects: Premenopausal studies		0.77	0.69–0.85

## 4 Discussion

The results of this trial-level meta-analysis demonstrated that, in the base case analysis (all five RCTs), AI ± OFS was associated with significantly improved DFS compared with TAM ± OFS, resulting in a 32 % reduction in risk of recurrence or death. These results were consistent in all scenario analyses conducted.

The results of our meta-analysis are consistent with several prior analyses. One prior meta-analysis of postmenopausal patients with endocrine receptor-positive EBC used individual patient data (from 31,920 patients) from nine RCTs and demonstrated that AI reduced recurrence by approximately 30 % compared with TAM (rate ratio, 0.70; 95 % CI, 0.64–0.77) [13]. A subsequent analysis of premenopausal patients included 7030 patients from four trials and demonstrated a significantly lower risk of recurrence with AI + OFS vs TAM + OFS (rate ratio for years 0–4, 0.68, 95 % CI, 0.55–0.85; *P* < .0001) [12]. Finally, in a recent real-world analysis of 2507 patients from a database of US academic and community oncology clinics (ConcertAI), most patients with stage II or III HR+/HER2− EBC were treated with NSAI ± OFS (74 %) vs TAM ± OFS (26 %) [14]. Using adapted invasive DFS (iDFS; based on STEEP criteria) as the endpoint, this analysis demonstrated an approximately 17 % reduction in relative risk of an iDFS event with NSAI ± OFS vs TAM ± OFS (hazard ratio, 0.83; 95 % CI, 0.69–0.98).

In our analysis, a greater benefit with AI over TAM was observed in premenopausal patients (AI + OFS vs TAM ± OFS) compared with postmenopausal patients (AI alone vs TAM alone). A number of RCTs (eg, SOFT/TEXT, ASTRRA, and STO-5) have shown the benefit of OFS in premenopausal patients, particularly those with high-risk HR+/HER2− EBC [15]. However, use of OFS in these patients is not consistent; recent clinical trials in patients with high-risk HR+/HER2− EBC included a substantial number of premenopausal patients who did not receive OFS with the ET partner [16].

Our study does have some limitations. First, the DFS hazard ratio data were used as reported in each publication without further modification. Definitions of DFS may have differed between each study, possibly leading to some heterogeneity that was unaccounted for in the analysis, although *I*^2^ = 0 was consistently observed for the base case and scenario analyses. In one of the trials (NCT01352091), the study design was such that patients were treated with TAM for 2–3 years before being randomized to anastrozole + goserelin or TAM for a total ET treatment duration of 5 years; this design may have impacted the generalizability and interpretation of the analyses that included NCT01352091. Additionally, while the SOFT trial included a TAM + OFS arm, no direct comparison was reported vs AI + OFS; therefore, this comparison (AI + OFS vs TAM + OFS) in SOFT was not included in the meta-analysis. Additionally, the TEXT trial was not included in the base case analysis because all reported DFS data were pooled with data from SOFT, and individual trial data were not available. However, a sensitivity analysis demonstrated results that were consistent with the base case and scenario analyses. Also, we could not assess whether the DFS benefit extended to mortality. Although OS is considered the gold standard of oncology clinical trials, we could not conduct a similar meta-analysis with OS as the endpoint due to limited data. However, it should be noted that a previous correlation analysis of 14 RCTs showed DFS as a reliable surrogate endpoint for OS in adjuvant HR+/HER2− EBC trials [17]. It should be noted there are data in clinical trials studying TAM + OFS vs AI + OFS that need consideration. In the SOFT trial, AI + OFS demonstrated reduction in risk of recurrence and mortality compared to TAM + OFS. However, analysis of patient subgroups seemed to suggest that the benefit of AI + OFS was greatest among patients with higher-risk disease. Decisions for AI + OFS or TAM + OFS would need to take into account factors like risk of recurrence and AE profiles of each treatment. TAM and AI (including combinations of AI with ovarian suppression) have different adverse event profiles that may impact choice of ET. A quality-of-life analysis among ET treatments was also not possible because differences in patient-reported outcome measures among the studies in the meta-analysis did not allow for such a comparison. Menopausal status definitions were also not consistent across trials. Premenopausal patients were identified using levels of estradiol, follicle stimulating hormone, and luteinizing hormone with additional specified criteria or using date of last menstrual period (NCT01352091, SOFT, HOBOE, SOFT, and BIG 1–98 [for exclusion of patients from trial]). The N-SAS BC03 trial included postmenopausal patients based on age or previous surgery (hysterectomy or ovariectomy). The different methods for determining menopausal status may have resulted in heterogeneity in outcomes and were not accounted for in our analysis.

## 5 Conclusion

Here, we report the results of a meta-analysis comparing AI ± OFS vs TAM ± OFS using disease-free survival data as published in 5 trials. OS was not included because of limited data in these trials. This meta-analysis demonstrated that AI ± OFS was associated with reduced risk of recurrence vs TAM ± OFS, including in the base case analysis, among NSAI-only treatments, and among both postmenopausal and premenopausal patients. These results were consistent with and support prior analyses that showed AI is the more effective ET vs TAM for adjuvant treatment of HR+/HER2− EBC.

## CRediT authorship contribution statement

**Wolfgang Janni:** Writing – review & editing, Conceptualization. **Michael Untch:** Writing – review & editing, Conceptualization. **Nadia Harbeck:** Writing – review & editing, Conceptualization. **Joseph Gligorov:** Writing – review & editing, Conceptualization. **William Jacot:** Writing – review & editing, Conceptualization. **Stephen Chia:** Writing – review & editing, Conceptualization. **Jean-François Boileau:** Writing – review & editing, Conceptualization. **Subhajit Gupta:** Writing – review & editing, Methodology, Formal analysis, Conceptualization. **Namita Mishra:** Writing – review & editing, Methodology, Formal analysis, Conceptualization. **Murat Akdere:** Writing – review & editing, Methodology, Formal analysis, Conceptualization. **Andriy Danyliv:** Writing – review & editing, Methodology, Formal analysis, Conceptualization. **Giuseppe Curigliano:** Writing – review & editing, Methodology, Formal analysis, Conceptualization.

## Data availability statement

Not applicable.

## Funding

This work was supported by 10.13039/100004336Novartis, which also provided financial support for medical editorial assistance.

## Disclosure of potential conflicts of interest

**W. Janni** reports personal fees from Amgen, AstraZeneca, Daiichi Sankyo, Lilly, MSD, Novartis, Pfizer, Roche, Seagen, and Gilead; employment from Universitätsklinikum Ulm; and speaker fees to institution from Novartis, GSK, Sanofi, Amgen, Roche, and Lilly; Prof Dr Janni serves as the chair of AGO Breast Council. **M. Untch** reports personal fees to institution from AstraZeneca, Amgen, Daiichi Sankyo, Lilly, Roche, Pfizer, MSD Oncology, Seagen, Pierre Fabre, Sanofi Aventis, Myriad, Gilead, Novartis, Stemline, Genzyme, and Medac. **N. Harbeck** reports personal fees and grants to institution from Novartis, Lilly, AstraZeneca, Daiichi Sankyo, MSD, Pierre Fabre, Roche, and Seagen; personal fees from Pfizer, Sandoz/Hexal, Gilead, Sanofi, Viatris, Medscape, Onkowissen, Zuellig Pharma, Aptitude Health, and Art Tempi; grants to institution from West German Study Group (WGSG), Palleos, TRIO, and BMS; and other funding from WGSG, AGO Breast Committee, ESO/ESCO, and BreastCare Journal. **J. Gligorov** reports grants from Roche-Genentech, Eisai, Exact Science, Pfizer, and Mylan; and personal fees and travel support from Roche-Genentech, Novartis, Daiichi Sankyo, MSD, Eisai, Exact Science, Pfizer, Mylan, Lilly, Pierre Fabre, and AstraZeneca. **W. Jacot** reports an institutional research grant, advisory board fees, and travel funding from AstraZeneca; advisory board fees and travel funding from Eisai, Novartis, Roche, Pfizer, Eli Lilly, Chugai, and Gilead; advisory board fees from MSD, BMS, and Seagen, and an institutional research grant and advisory board fees from Daichi Sankyo. **S. Chia** reports personal fees and funds to institution from Novartis, Pfizer, F. Hoffman-LaRoche, Eli Lilly, Merck, and AstraZeneca. **J-F. Boileau** reports personal fees and institution principal investigator study funding from Novartis, AstraZeneca, Pfizer, Roche, Merck, Lilly, and Bristol Myers Squibb. **S. Gupta, N. Mishra, M. Akdere,** and **A. Danyliv** report employment with and stock ownership in Novartis. **G. Curigliano** reports personal fees from Roche, Novartis, Lilly, Pfizer, AstraZeneca, Daichii Sankyo, Ellipsis, Veracyte, Exact Science, Celcuity, Merck, BMS, Gilead, Sanofi, and Menarini.

## References

[1] Howlader N., Altekruse S.F., Li C.I., Chen V.W., Clarke C.A., Ries L.A., Cronin K.A. (2014). US incidence of breast cancer subtypes defined by joint hormone receptor and HER2 status. J Natl Cancer Inst.

[2] Pistilli B., Lohrisch C., Sheade J., Fleming G.F. (2022). Personalizing adjuvant endocrine therapy for early-stage hormone receptor-positive breast cancer. Am Soc Clin Oncol Educ Book.

[3] Zhao H., Lei X., Niu J., Zhang N., Duan Z., Chavez-MacGregor M., Giordano S.H. (2021). Prescription patterns, initiation, and 5-Year adherence to adjuvant hormonal therapy among commercially insured patients with breast cancer. JCO Oncology Practice.

[4] Gremke N., Griewing S., Chaudhari S., Upadhyaya S., Nikolov I., Kostev K., Kalder M. (2023). Persistence with tamoxifen and aromatase inhibitors in Germany: a retrospective cohort study with 284,383 patients. J Cancer Res Clin Oncol.

[5] Ferreira A.R., Palha A., Correia L., Filipe P., Rodrigues V., Miranda A. (2018). Treatment adoption and relative effectiveness of aromatase inhibitors compared to tamoxifen in early breast cancer: a multi-institutional observational study. Breast.

[6] Criscitiello C., Spurden D., Rider A., Williams R., Corsaro M., Pike J., Law E.H. (2020).

[7] Record H., Clelland E., Rothschild H.T., Kaur M., Chien A.J., Melisko M. (2023). Tamoxifen or aromatase inhibitors with ovarian function suppression in pre-menopausal stage I-III lobular breast cancer. npj Breast Cancer.

[8] Howell A. (2005). ATAC trial update. Lancet.

[9] Pagani O., Regan M.M., Walley B.A., Fleming G.F., Colleoni M., Láng I. (2014). Adjuvant exemestane with ovarian suppression in premenopausal breast cancer. N Engl J Med.

[10] Francis P.A., Pagani O., Fleming G.F., Walley B.A., Colleoni M., Láng I. (2018). Tailoring adjuvant endocrine therapy for premenopausal breast cancer. N Engl J Med.

[11] Dowsett M., Cuzick J., Ingle J., Coates A., Forbes J., Bliss J. (2010). Meta-analysis of breast cancer outcomes in adjuvant trials of aromatase inhibitors versus tamoxifen. J Clin Oncol.

[12] Bradley R., Braybrooke J., Gray R., Hills R.K., Liu Z., Pan H. (2022). Aromatase inhibitors versus tamoxifen in premenopausal women with oestrogen receptor-positive early-stage breast cancer treated with ovarian suppression: a patient-level meta-analysis of 7030 women from four randomised trials. Lancet Oncol.

[13] Early Breast Cancer Trialists' Collaborative Group (EBCTCG) (2015). Aromatase inhibitors versus tamoxifen in early breast cancer: patient-level meta-analysis of the randomised trials. Lancet.

[14] Graff S.L., Tolaney S.M., Hart L.L., Razavi P., Fasching P.A., Janni W. (2023). Real-world outcomes with adjuvant nonsteroidal aromatase inhibitors (NSAIs) vs tamoxifen (TAM) in patients with hormone receptor−positive/human epidermal growth factor receptor 2−negative (HR+/HER2−) early breast cancer (EBC): a US database analysis. J Clin Oncol.

[15] Ma L., Yang B., Wu J. (2024). Revisiting ovarian function suppression with GnRH agonists for premenopausal women with breast cancer: who should use and the impact on survival outcomes. Cancer Treat Rev.

[16] Paluch-Shimon S., Neven P., Huober J., Cicin I., Goetz M.P., Shimizu C. (2023). Efficacy and safety results by menopausal status in monarchE: adjuvant abemaciclib combined with endocrine therapy in patients with HR+, HER2-, node-positive, high-risk early breast cancer. Ther Adv Med Oncol.

[17] Untch M., Pérol D., Mayer E.L., Cortes J., Nusch A., Cameron D. (2024). Disease-free survival as a surrogate for overall survival in HR+/HER2- early breast cancer: a correlation analysis. Eur J Cancer.

