# Supplementary material for: Systematic literature review and trial-level meta-analysis of aromatase inhibitors vs tamoxifen in patients with HR+/HER2− early breast cancer
Source: Breast. 2025 Mar 5;81:104429. doi: 10.1016/j.breast.2025.104429 (PMC11992420; doi:10.1016/j.breast.2025.104429)
Supplement: Multimedia component 1 [file mmc1.docx]

**Appendix**

**Supplementary Methods**

A systematic literature review (SLR) was conducted using biomedical databases (Embase, Medline, and Cochrane) and data from key conferences during the period from database inception to March 24, 2023. This SLR was updated with the most recent evidence (database searches until October 2023 and conferences until December 2023). The SLR was conducted in accordance with the Cochrane Handbook for Systematic Reviews of Interventions and the Preferred Reporting Items for Systematic Reviews and Meta-Analyses (PRISMA) guidelines (<https://www.prisma-statement.org/>).

A key inclusion criteria for the SLR were studies that included the HR+/HER2− EBC population. Therefore, we included studies that enrolled only patients with HR+/HER2− EBC, studies with ≥80% with HR+/HER2− EBC, or studies that had <80% HR+/HER2− disease but reported results in the HR+/HER2− subgroup. Studies with <80% of patients having HR+/HER2− tumors and not providing subgroup results were excluded. Publications in which the HER2 status was unclear (possibly due to the unavailability of testing during the conduct of the study) were excluded.

A total of 44 studies reported in 136 publications were returned through the SLR from which a final five RCTs (SOFT, HOBOE, BIG 1-98, N-SAS BC03, and NCT01352091) were identified for inclusion in the meta-analysis by meeting the following criteria: phase 3 RCTs, studies with a direct comparison between aromatase inhibitors (AIs) and tamoxifen, and studies reporting a disease-free survival (DFS) hazard ratio.

**Supplemental Figure 1. PRISMA Diagram for SLR**


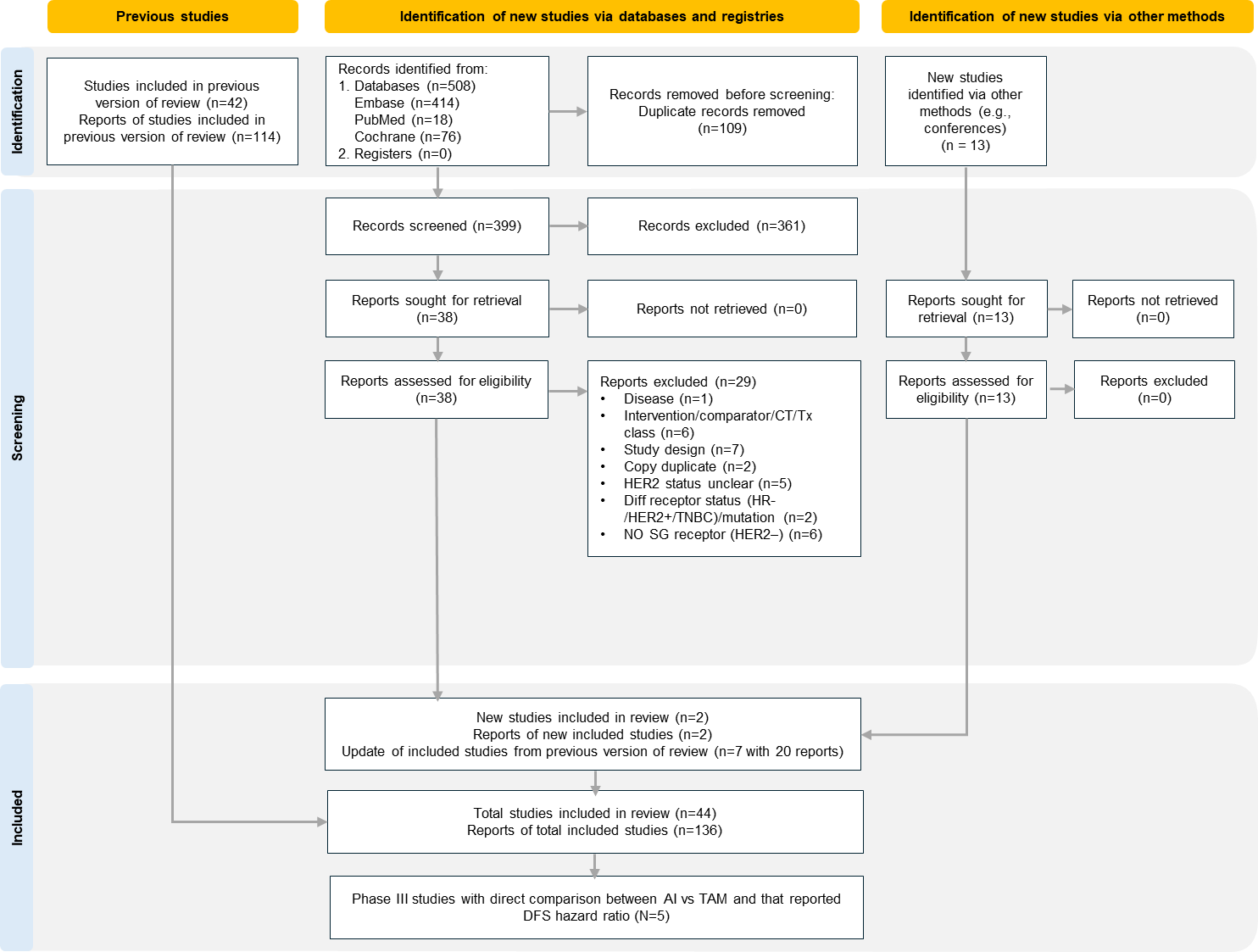


DFS, disease-free survival; HER2, human epidermal growth factor receptor 2; HR, hormone receptor; SG, subgroup; SLR, systematic literature review; TAM, tamoxifen; TNBC, triple-negative breast cancer.

**
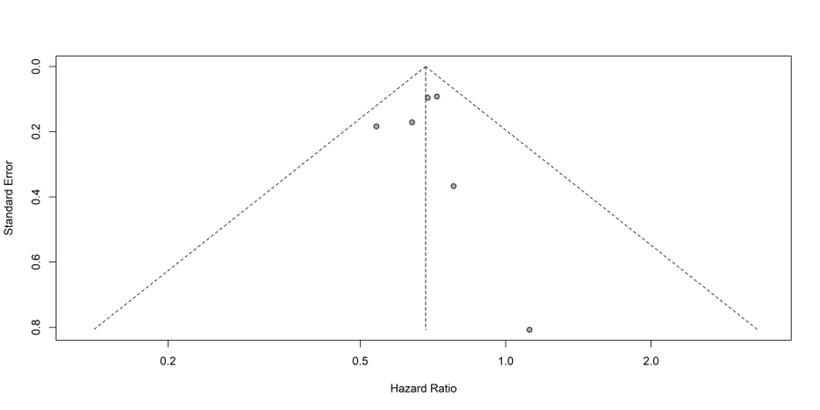
Supplemental Figure 2. Funnel Plot of All Trials Included in the Meta-Analysis**

**Supplemental Table 1. Patients and Disease Characteristics in Included Trials (HR+/HER2- Subgroups)**

| **Trial** | **Arm** | | **Median Age (IQR)** | **Nodal status** | **Tumor size** | **Tumor grade** | **Prior Chemo** |
| --- | --- | --- | --- | --- | --- | --- | --- |
| SOFT | Exemestane + OFS | | NA | NA | NA | NA | NA |
|  | TAM | | 43  (38-46) | N0: 65.0%  N1: 25.3%  N2: 9.6% | <2 cm: 66.4%  2-5 cm: 27.5%  ≥5 cm: 3.4% | G1: 27.0%  G2: 48.3%  G3: 22.3% | NA |
| HOBOE | Letrozole + OFS | | 44.9  (40.8-48.0) | N0: 55.1%  N1: 30.6%  N2: 10.7%  N3: 3.7% | NA | G1: 9.3%  G2: 49.7%  G3: 36.0% | Neoadjuvant: 62.6% |
|  | TAM + OFS | | 44.7  (41.3-48.0) | N0: 54.5%  N1: 31.4%  N2: 9.6%  N3: 4.5% | NA | G1: 10.2%  G2: 55.1%  G3: 31.6% | Neoadjuvant: 62.7% |
| HOBOE | Zoledronic acid + letrozole + OFS | | 45.2  (40.9-48.1) | N0: 54.6%  N1: 31.0%  N2: 9.9%  N3: 4.5% | NA | G1: 7.3%  G2: 57.5%  G3: 33.0% | Neoadjuvant: 62.5% |
|  | TAM + OFS | | 44.7  (41.3-48.0) | N0: 54.5%  N1: 31.4%  N2: 9.6%  N3: 4.5% | NA | G1: 10.2%  G2: 55.1%  G3: 31.6% | Neoadjuvant: 62.7% |
| BIG 1-98 | Letrozole | Lobular | NA | N0: 54.6%  N1: 25.7%  N2: 19.7% | <2 cm: 48.0%  2-5 cm: 51.3% | G1: 5.3%  G2: 94.7%  G3: NA | Neoadjuvant: 27.0% |
|  |  | Ductal | NA | N0: 56.9%  N1: 27.5%  N2:15.6% | <2 cm: 65.8%  2-5 cm: 33.9% | G1: 20.6%  G2: 56.1%  G3: 22.8% | Neoadjuvant: 20% |
|  | TAM | Lobular | NA | N0: 55.2%  N1: 25.6%  N2: 19.2% | <2 cm: 49.4%  2-5 cm: 48.8% | G1: 2.9%  G2: 95.9%  G3: 1.2% | Neoadjuvant: 26.7% |
|  |  | Ductal | NA | N0: 56.4%  N1: 27.1%  N2: 16.5% | <2 cm: 63.9%  2-5 cm: 35.7% | G1: 21.9%  G2: 55.0%  G3: 22.6% | Neoadjuvant: 21.6% |
| N-SAS BC03 | Anastrozole | | NA | NA | NA | NA | NA |
|  | TAM | | NA | NA | NA | NA | NA |
| NCT01352091 | TAM 🡪 goserelin + anastrozole | | 41  (range: 32–50) | N0: 12.1%  N1: 51.5%  N2: 27.3%  N3: 9.1% | NA | G1: 0  G2: 60.6%  G3: 9.1% | Neoadjuvant:18.2% |
|  | TAM | | 41  (range: 29–51) | N0: 7.1%  N1: 75.0%  N2: 14.3%  N3: 3.6% | NA | G1: 0  G2: 62.1%  G3: 6.9% | Neoadjuvant:20.7% |

NA, not available for the HR+/HER2- subgroup.

**Supplemental Table 2. Risk of Bias Assessment**

| **Trial** | **Was the randomization carried out appropriately? (Yes/No/Not clear/NA)** | **Was the concealment of treatment allocation adequate? (Yes/No/Not clear/NA)** | **Were the groups similar at the outset of the study in terms of prognostic factors? (Yes/No/Not clear/NA)** | **Were the care providers, participants and outcome assessors blind to treatment allocation? (Yes/No/Not clear/NA)** | **Were there any unexpected imbalances in drop-outs between groups? (Yes/No/Not clear/NA)** | **Is there any evidence to suggest that the authors measured more outcomes than they reported? (Yes/No/Not clear/NA)** | **Did the analysis include an intention-to-treat analysis? If so, was this appropriate and were appropriate methods used to account for missing data? (Yes/No/Not clear/NA)** | **Risk of bias (Low/High/Unclear)** |  |
| --- | --- | --- | --- | --- | --- | --- | --- | --- | --- |
| **SOFT** | Yes. All eligible patients were randomized via a centralized service (Trans European Network for Clinical Trials Services [TENALEA]) | Yes. Randomization was performed by means of the IBCSG Internet-based system, with the use of permuted blocks | Yes. The treatment groups were well balanced at baseline | No. This was an open-label study | No. There were no unexpected imbalances in drop-outs between groups. The reasons for withdrawals were clearly reported and comparable across the treatment groups. | No. Authors measured all the pre-specified outcomes and no evidence of measurement of more outcomes was observed | Yes. ITT analysis was used for efficacy outcomes and mITT analysis was used for safety outcomes. | Low risk |  |
| **HOBOE** | Yes. All eligible patients were randomized via web-based trial platform | Yes. Randomization was performed via the web-based trial platform at the Clinical Trials Unit of the Istituto Nazionale Tumori, Napoli, Italy. | Yes. The treatment groups were well balanced at baseline | No. This was an open-label study | No. There were no unexpected imbalances in drop-outs between groups. The reasons for withdrawals were clearly reported and comparable across the treatment groups. | No. Authors measured all the pre-specified outcomes and no evidence of measurement of more outcomes was observed | Yes. ITT analysis was used for efficacy outcomes and PP analysis was used for safety outcomes. | Low risk |  |
| **BIG 1-98** | Yes. All eligible patients were randomized but method was unclear | Not clear. | Yes. The treatment groups were well balanced at baseline | Yes. This was a double-blind study | Not clear. | No. Authors measured all the pre-specified outcomes and no evidence of measurement of more outcomes was observed | Yes. ITT analysis was used for efficacy outcomes | Unclear |  |
| **N-SAS BC03** | Yes. All eligible patients were randomized but method was unclear | Yes. Eligible patients were randomly assigned to continue tamoxifen or to switch to anastrozole in a ratio of 1:1 according to the allocation adjustment factors, using a dynamic allocation method. | Yes. The treatment groups were well balanced at baseline | No. This was an open-label study | No. There were no unexpected imbalances in drop-outs between groups. The reasons for withdrawals were clearly reported and comparable across the treatment groups. | Yes. Authors measured HRQoL however outcomes for that was not reported | Yes. mITT analysis was used for efficacy and safety outcomes. | Unclear |  |
| **NCT01352091** | Yes. Randomization was performed by filling in randomization forms and faxing them to the data processing center. The patients were randomly assigned via random numbers to the treatment group by the designated service provider using an Interactive Voice Response System. | Yes. Centralized process was adapted | Yes. The treatment groups were well balanced at baseline | No. This was an open-label study | No. There were no unexpected imbalances in drop-outs between groups. The reasons for withdrawals were clearly reported and comparable across the treatment groups. | No. Authors measured all the pre-specified outcomes and no evidence of measurement of more outcomes was observed | Yes. ITT analysis was used for efficacy and safety outcomes. | Low risk |  |
